# Adherence to Guidelines for Cancer Survivors and Health-Related Quality of Life among Korean Breast Cancer Survivors

**DOI:** 10.3390/nu7125532

**Published:** 2015-12-09

**Authors:** Sihan Song, Eunkyung Hwang, Hyeong-Gon Moon, Dong-Young Noh, Jung Eun Lee

**Affiliations:** 1 Department of Food and Nutrition, Sookmyung Women’s University, Cheonpa-ro 47-gil 100, Yongsan-gu, Seoul 140-742, Korea; songsihan@sm.ac.kr; 2 Breast Care Center, Seoul National University Hospital, 103 Daehak-ro, Jongno-gu, Seoul 110-744, Korea; gogh001@hanmail.net (E.H.); moonhg74@snu.ac.kr (H.-G.M.); dynoh@snu.ac.kr (D.-Y.M.); 3 Department of Surgery and Cancer Research Institute, Seoul National University College of Medicine, 103 Daehak-ro, Jongno-gu, Seoul 110-744, Korea

**Keywords:** breast cancer survivors, cancer survivor guidelines, health-related quality of life

## Abstract

There is limited evidence on the association between adherence to guidelines for cancer survivors and health-related quality of life (HRQoL). In a cross-sectional study of Korean breast cancer survivors, we examined whether adherence to the guidelines of the American Cancer Society (ACS) and World Cancer Research Fund/American Institute for Cancer Research (WCRF/AICR) for cancer survivors was related to levels of HRQoL, assessed by the Korean version of Core 30 (C30) and Breast cancer module 23 (BR23) of the European Organization for Research and Treatment of Cancer-Quality of Life Questionnaire (EORTC-QLQ). We included a total of 160 women aged 21 to 79 years who had been diagnosed with breast cancer according to American Joint Committee on Cancer (AJCC) stages I to III and had breast cancer surgery at least six months before the interview. Increasing adherence to ACS guidelines was associated with higher scores of social functioning (*p* for trend = 0.05), whereas increasing adherence to WCRF/AICR recommendations was associated with higher scores of arm symptoms (*p* for trend = 0.01). These associations were limited to those with stage II or III cancer. Diet may be an important factor in relation to quality of life among Korean breast cancer survivors, however our findings warrant further prospective studies to evaluate whether healthy diet improves survivors’ quality of life.

## 1. Introduction

Breast cancer is the most frequent cancer among women worldwide [1]; nonetheless, early detection and advances in breast cancer treatment have continued to contribute to a decline in breast cancer mortality [2,3]. In Korea, the age-standardized incidence rate of breast cancer was 10.7 per 100,000 in 1999, which increased to 22.6 per 100,000 in 2012, with a 6.0% average annual percentage change and becoming the second most commonly diagnosed cancer among women [4]. From 2008 to 2012, the five-year survival rate of breast cancer patients was 91.3% [4]. This dramatic increase in breast cancer incidence and the high survival rate among Korean breast cancer patients indicates the importance of health-related quality of life (HRQoL) and its relationship with lifestyle, including diet and exercise [5].

Evidence regarding how breast cancer survivors try to improve their prognosis is limited to date. In 2007, the World Cancer Research Fund/American Institute for Cancer Research (WCRF/AICR) advised that cancer survivors should follow recommendations for cancer prevention [6]. For breast cancer survivors, in 2014, the Continuous Update Project of the World Cancer Research Fund concluded that there was limited evidence for the specific recommendations for breast cancer survivors and its advice was to follow WCRF/AICR cancer prevention recommendations [7]. In 2012, the American Cancer Society (ACS) released “Nutrition and Physical Activity Guidelines for Cancer Survivors”, providing guidelines on body weight, physical activity and diet, in which the dietary guideline was based on the ACS guidelines for the prevention of cancer [8].

A recent review of epidemiological studies on breast cancer survivorship reported that high body fat and most likely low physical activity increase the risk of breast cancer mortality; however, the association of dietary factors with cancer survivorship was unclear [9]. Several prospective cohort studies have suggested the potential link between adherence to guidelines for cancer prevention and breast cancer incidence among the cancer-free population [10,11,12,13,14]. However, only a few studies have explored whether adherence to these guidelines improves quality of life or prognosis among breast cancer survivors. The ACS’s Study of Cancer Survivors-II showed that cancer survivors, including breast cancer survivors, who followed the recommendations for physical activity, fruit and vegetable consumption and smoking, had significantly higher HRQoL scores [15]. For cancer survivors in general, some studies reported that adherence to WCRF/AICR guidelines for cancer prevention was associated with a lower risk of death [16] and higher levels of HRQoL [17] among female cancer survivors.

Although healthy lifestyle may benefit Korean breast cancer survivors, evidence is limited. Therefore, we examined whether adherence to lifestyle behavior recommendations was associated with HRQoL levels among Korean breast cancer survivors.

## 2. Materials and Methods

### 2.1. Study Population

Study participants who had been diagnosed with breast cancer according to the American Joint Committee on Cancer (AJCC) Cancer Staging Manual were enrolled at University Hospital in Seoul between September 2012 and July 2014. Two-hundred and nineteen women aged 21 to 79 years signed an informed consent form. We included participants who had been diagnosed with invasive primary breast cancer at AJCC stages I to III and had breast cancer surgery at least 6 months before the interview. We excluded the following respondents: women who had breast cancer surgery less than 6 months before the interview (*n* = 9), women who had been diagnosed with stage 0 breast cancer (*n* = 6), women who had metastasis (*n* = 14), women who had been diagnosed with other cancers before the interview of our study (*n* = 16), of whom medical records were missing (*n* = 17) or women who did not appropriately complete dietary records (*n* = 5). As a result, a total of 160 women were eligible for this study. The institutional review board at Seoul National University Hospital, Seoul, Korea, approved this study.

### 2.2. Data Collection

Participants were surveyed by well-trained nurses using a structured questionnaire. Data on height and weight, socio-demographic status, smoking status, alcohol intake, physical activity, HRQoL, reproductive history, and use of dietary supplements were collected. Clinical features including T stage (tumor), N stage (node), M stage (metastasis), and hormone receptor status were obtained from hospital medical records. The participants recorded their food intake for 2 weekdays and 1 weekend day. Body mass index was calculated as weight (kg) divided by the square of height (m^2^). We obtained information about the duration and frequency of post-diagnosis physical activity and calculated the scores for metabolic equivalent task (MET) hours per week by multiplying the hours per week engaged in that activity by the activity’s corresponding MET value [18,19]. The scores of MET-hours per week for each activity were summed to calculate a total MET-hours per week score. We calculated the energy and nutrient intake of the participants using the Computer-Aided Nutritional Analysis Program (CAN-Pro) 4.0 (Korean Nutrition Information Center, Seoul, Korea)*.*

### 2.3. Operationalization of ACS Guidelines

The ACS guidelines for cancer survivors are as follows: (1) achieving and maintaining a healthy body weight; (2) adopting regular physical activity; and (3) achieving a dietary pattern that is high in vegetables, fruits, and whole grains [8]. The scoring criteria of the ACS guidelines are presented in Table 1. For BMI classification of the Asia-Pacific region [20], we assigned 0, 1 and 2 to <18.5 or >25, 23 to 25, and 18.5 to 22.9 kg/m^2^ of BMI, respectively. We grouped participants into three groups based on tertiles of adherence levels for physical activity (MET-hour/week), fruit and vegetable intake (g/day), proportion of whole grain to total grain intake (%), and red and processed meat intake (g/day). The highest adherence levels were given a score of 2, middle levels were given a score of 1, and the lowest levels were given a score of 0. Because the third guideline includes three sub-guidelines, in order to treat the three guidelines the same, we regrouped the participants into three categories of 0, 1, and 2 based on tertiles of the sum of the three adherence scores of the sub-guidelines. We calculated the overall adherence scores by summing the three scores of adherence to the ACS guidelines (range 0–6).

**Table 1:** Operationalization of adherence to ACS guidelines.

ACS Guidelines for Cancer Survivors	Sub-Guidelines	Operationalization	Scoring	
1. Achieve and maintain a healthy weight.	If overweight or obese, limit consumption of high-calorie foods and beverages and increase physical activity to promote weight loss.	BMI (kg/m^2^)		
		18.5–22.9	2	
		23-25	1	
		<18.5 or >25	0	
2. Engage in regular physical activity.	Avoid inactivity and return to normal daily activities as soon as possible following diagnosis. Aim to exercise at least 150 min per week. Include strength training exercises at least 2 days per week.	METs (h/week)		
		Tertile 3 (>40.3)	2	
		Tertile 2 (19.6–43.3)	1	
		Tertile 1 (<19.6)	0	
3. Achieve a dietary pattern that is high in vegetables, fruits, and whole grains.	(3a) Eat 5 or more servings of a variety of vegetables and fruits each day.	Fruits and vegetables intake (g/day)		
		Tertile 3 (>659.2)	2	Tertiles High 2 Middle 1 Low 0
		Tertile 2 (443.1–659.2)	1	
		Tertile 1 (<443.1)	0	
	(3b) Choose whole grains in preference to processed (refined) grains.	Percentage of grains consumed as whole grains (%)		
		Tertile 3 (90–100)	2	
		Tertile 2 (64.3–90)	1	
		Tertile 1 (0–64.3)	0	
	(3c) Limit consumption of processed and red meats.	Red and processed meat intake (g/day)		
		Tertile 1 (<5)	2	
		Tertile 2 (5–60.7)	1	
		Tertile 3 (>60.7)	0	

Abbreviations: ACS, American Cancer Society; BMI, Body Mass Index; MET, metabolic equivalent task.

### 2.4. Operationalization of WCRF/AICR Recommendations

The WCRF/AICR has released 10 recommendations including 2 special recommendations for cancer prevention [6]. We constructed adherence scores using the following 6 recommendations: (1) be as lean as possible within the normal range of body weight; (2) be physically active as part of everyday life; (3) limit consumption of energy-dense foods and avoid sugary drinks; (4) eat mostly foods of plant origin; (5) limit intake of red meat and avoid processed meat; and (6) limit consumption of salt and avoid moldy cereals (grains) or pulses (legumes). We excluded the parts on alcohol consumption and dietary supplement use because of mixed findings for the association between alcohol drinking [21,22,23,24] or supplement use [25,26] and breast cancer recurrence or death among breast cancer survivors. We did not include the breastfeeding recommendation because previous studies on breast cancer mortality have reported inconsistent results [27,28,29].

The scoring criteria of the WCRF/AICR recommendations are presented in Table 2. For BMI classification of the Asia-Pacific region [20], we assigned 0, 1 and 2 to <18.5 or >25, 23 to 25, and 18.5 to 22.9 kg/m^2^ of BMI, respectively. We grouped the participants into three groups based on tertiles of adherence levels for physical activity (MET-hour/week), energy-dense food intake (kcal/100 g), non-starchy vegetable and fruit intake (g/day), refined grain intake (g/day), red and processed meat intake (g/day), and sodium intake (mg/day). The highest adherence levels were given a score of 2, middle levels were given a score of 1, and the lowest levels were given a score of 0. We assigned 0, 1 and 2 to >50, 0–50, and 0 g/day of sugary drinks, respectively. Because the third and fourth guidelines include two sub-guidelines, to treat the scoring the same, we regrouped the participants into three categories of 0, 1, and 2 based on tertiles of the sum of two adherence scores of the sub-guidelines of the third or fourth guidelines. We calculated the overall adherence scores by summing the six scores of adherence to the WCRF/AICR guidelines (range 0–12).

**Table 2:** Operationalization of adherence to WCRF/AICR recommendations.

WCRF/AICR Recommendations for Cancer Survivors	Sub-Recommendations	Operationalization	Scoring	
1. Body fatness: Be as lean as possible without becoming underweight	(1a) Ensure that body weight throughout childhood and adolescent growth projects toward the lower end of the normal BMI range at age 21 years (1b) Maintain body weight within the normal range from age 21 years (1c) Avoid weight gain and increases in waist circumference throughout adulthood	BMI (kg/m^2^)		
		18.5–22.9	2	
		23-25	1	
		<18.5 or >25	0	
2. Physical activity: Be physically active as part of your everyday life	(2a) Be moderately physically active, equivalent to brisk walking, for ≥30 min every day (2b) As fitness improves, aim for ≥60 min of moderate, or for ≥30 min of vigorous physical activity every day (2c) Limit sedentary habits such as watching television	Physical activity (MET-hour/week)		
		Tertile 3 (>40.3)	2	
		Tertile 2 (19.6–43.3)	1	
		Tertile 1 (<19.6)	0	
3. Foods and drinks that promote weight gain: Limit consumption of energy-dense foods; avoid sugary drinks	(3a) Consume energy-dense foods (225–275 kcal/100 g) sparingly (3b) Avoid sugary drinks (servings/week) (3c) Consume fast foods sparingly, if at all	Energy dense diet ^1^ (kcal/100 g)		Tertiles High 2 Middle 1 Low 0
		Tertile 1 (<154.2)	2	
		Tertile 2 (132–154.2)	1	
		Tertile 3 (>132)	0	
		Sugary drinks (g/day)		
		None	2	
		≤50	1	
		>50	0	
4. Plant foods: Eat mostly foods of plant origin	(4a) Eat ≥5 portions/servings (>400 g) of a variety of non-starchy vegetables and of fruits every day (4b) Eat relatively unprocessed cereals (grains) and/or pulses (legumes) with every meal (4c) Limit refined starchy foods (4d) People who consume starchy roots or tubers as staples should also ensure sufficient intake of non-starchy vegetables, fruit, and pulses (legumes)	Non-starchy vegetable and fruit intake (g/day)		Tertiles High 2 Middle 1 Low 0
		Tertile 3 (>586.2)	2	
		Tertile 2 (371.5–586.2)	1	
		Tertile 1 (<371.5)	0	
		Refined grains (g/day)		
		Tertile 1 (<15)	2	
		Tertile 2 (15–45)	1	
		Tertile 3 (>45)	0	
5. Animal foods: Limit intake of red meat (beef, pork, lamb, and goat) and avoid processed meat	(5a) People who eat red meat should consume <500 g/week, very little if any to be processed meats	Red meat and processed meat intake (g/day)		
		Tertile 1 (<5)	2	
		Tertile 2 (5–60.5)	1	
		Tertile 3 (>60.5)	0	
6. Preservation, processing, preparation: Limit consumption of salt. Avoid moldy cereals (grains) or pulses (legumes)	(6a) Avoid salt-preserved, salted, or salty foods; preserve foods without using salt (6b) Limit consumption of processed foods with added salt to ensure an intake of<6 g (2.4 g sodium)/day (6c) Do not eat moldy cereals (grains) or pulses (legumes)	Sodium intake (mg/day)		
		Tertile 1 (<3521.7)	2	
		Tertile 2 (3521.7–4602.2)	1	
		Tertile 3 (>4602.2)	0	

Abbreviations: WCRF/AICR, World Cancer Research Fund/American Institute for Cancer Research; BMI, Body Mass Index; MET, metabolic equivalent task; ^1^ Energy dense diet denotes energy intake per amount of total food intake (kcal per 100 g).

### 2.5. Health-Related Quality of Life Measurement

We assessed HRQoL in breast cancer survivors using a validated Korean version of European Organization for Research and Treatment of Cancer Quality of Life Questionnaire Core 30 (EORTC QLQ-C30) version 3.0 and Quality of Life Questionnaire Breast Cancer Module 23 (QLQ-BR23) [30,31]. The QLQ-C30, developed for assessing cancer survivors HRQoL in international clinical trials, is composed of 30 items, with subcategories of global health status/quality of life (QoL) scale, functional scales (physical, role, emotional, cognitive, and social) and symptom scales (fatigue, nausea and vomiting, pain, dyspnea, insomnia, appetite loss, constipation, diarrhea, and financial difficulty). The QLQ-BR23 is a questionnaire for breast cancer survivors with regard to disease stage and treatment modality. The breast cancer module incorporates four functional scales (body image, sexual functioning, sexual enjoyment, and future perspective) and four symptom scales (systemic therapy side effects, breast symptoms, arm symptoms, and upset by hair loss) [32]. Sexual enjoyment was not included in this analysis because 70% of the participants did not respond to this domain. According to the EORTC scoring manual, we transformed raw scores, including 4-point or 7-point scales, to a 0 to 100 scale [33]. A higher score represents a higher (“better”) level of functioning and global health status /QoL or a higher (“worse”) level of symptoms or problems.

### 2.6. Statistical Analyses

Socio-demographic, lifestyle and clinical characteristics of the study participants are presented as the mean or frequency according to quartiles of adherence scores to ACS or WCRF/AICR guidelines. To examine the association between adherence scores to ACS or WCRF/AICR guidelines and HRQoL levels, we calculated the least square means (LS means) and 95% confidence intervals (95% CIs) using generalized linear models (GLMs).

We log-transformed variables and exponentiated them if they did not meet normality. To test for trends, we assigned the median values of the quartiles of adherence scores and treated the variable as a continuous variable. In the multivariate-adjusted models, we adjusted for age (year), energy intake (kcal/day), dietary supplement use (yes, no), marital status (married or cohabitation, unmarried or divorced or widowed), education level (high school or below, college or above), stage at diagnosis (I, II, and III), and time since surgery (6 months to 1 year, 1 year to 5 years, 5 years or more). We also conducted subgroup analyses to examine the associations by breast cancer stage at diagnosis (I, II or III) or time since surgery (<1.9 or ≥1.9 years, median). We grouped stage II and III because of the small number of breast cancer survivors (*n* = 17) with stage III. The statistical tests were two-sided, and *p* < 0.05 was considered statistically significant. All analyses were conducted using the SAS software 9.3.

## 3. Results

A total of 160 breast cancer survivors were included in our study. The ACS guideline score ranged from zero to six, and the WCRF/AICR recommendation score ranged from one to 12. The mean (SD) age of the study participants was 50.96 (8.72) years (range, 21–79 years). Of the study participants, 83.75% had undergone breast cancer surgery less than five years before the interview. The mean (SD) of physical activity level was 37.88 (37.78) METs-hour/week, and the mean (SD) of BMI was 22.58 (2.84) kg/m^2^. In total, 42.50% of the participants had graduated from college or an education above. Most of them were married (84.38%), and never smokers (86.88%). The prevalences of breast cancer stages I, II, and III were 45.63%, 43.75%, and 10.63%, respectively (Table 3). Breast cancer survivors in the highest quartile of ACS guideline scores or WCRF/AICR recommendation scores had lower BMI, and higher physical activity compared to those in the lowest quartile (Table 3).

Breast cancer survivors who had high adherence to ACS guidelines tended to have higher social functioning scores (*p* for trend = 0.05) compared to those with lower adherence to ACS guidelines (Table 4). When we analyzed according to the breast cancer stage at diagnosis (stages I and II–III), social functioning scores tended to rise with increasing scores of adherence to ACS guidelines (*p* for trend = 0.05), and sexual functioning scores significantly increased with increasing scores of adherence to ACS guidelines (*p* for trend = 0.01) among those with stages II or III (Table S2). Among 73 breast cancer survivors who had been diagnosed with stage I breast cancer, physical functioning was significantly associated with increasing scores of adherence to ACS guidelines (*p* for trend = 0.01) (Table S1).

**Table 3:** Characteristics of study participants according to adherence score of ACS or WCRF/AICR guidelines.

		ACS Guidelines Score		WCRF/AICR Recommendation Score	
Characteristic	All (*n* = 160)	Q1 (*n* = 50)	Q4 (*n* = 48)	Q1 (*n* = 40)	Q4 (*n* = 33)
Age, mea*n* ± SD (year)	50.96 ± 8.72	51.78 ± 6.84	52.75 ± 8.45	50.50 ± 9.09	50.76 ± 8.54
Body mass index, mea*n* ± SD (kg/m^2^)	22.58 ± 2.84	24.19 ± 3.57	21.25 ± 1.39	24.36 ± 3.44	21.41 ± 1.50
Physical activity, mea*n* ± SD (MET-hour/week)	37.88 ± 37.78	16.19 ± 22.74	58.07 ± 40.16	15.26 ± 11.59	52.56 ± 34.67
Educatio*n* level, *n* (%) ^1^					
High school or below	90 (56.25)	25 (50.00)	29 (60.42)	20 (50.00)	17 (51.52)
College or above	68 (42.50)	25 (50.00)	18 (37.50)	20 (50.00)	15 (45.45)
Marital status, *n* (%) ^1^					
Married or cohabitation	135 (84.38)	41 (82.00)	40 (83.33)	30 (75.00)	29 (87.88)
Unmarried or divorced or widowed	24 (15.00)	9 (18.00)	8 (16.67)	10 (25.00)	4 (12.12)
Current menopausal status, *n* (%) ^1^					
Premenopausal	16 (10.00)	5 (10.00)	1 (2.08)	5 (12.50)	3 (9.09)
Postmenopausal	137 (85.63)	43 (86.00)	44 (91.67)	35 (87.50)	29 (87.88)
Time since surgery, *n* (%)					
6 month–<1 year	26 (16.25)	8 (16.00)	8 (16.67)	7 (17.50)	3 (9.09)
1 year–<2 years	65 (40.63)	17 (34.00)	24 (50.00)	15 (37.50)	18 (54.55)
2 years–<5 years	43 (26.88)	14 (28.00)	11 (22.92)	9 (22.50)	8 (24.24)
≥5 years	26 (16.25)	11 (22.00)	5 (10.42)	9 (22.50)	4 (12.12)
AJCC stage at diagnosis, *n* (%)					
I	73 (45.63)	21 (42.00)	24 (50.00)	20 (50.00)	16 (48.48)
II	70 (43.75)	22 (44.00)	19 (39.58)	14 (35.00)	15 (45.45)
III	17 (10.63)	7 (14.00)	5 (10.42)	6 (15.00)	2 (6.06)
Estroge*n* receptor status, *n* (%) ^1^					
Negative	49 (30.63)	14 (28.00)	17 (35.42)	8 (20.00)	12 (36.36)
Positive	108 (67.50)	34 (68.00)	30 (62.50)	31 (77.50)	21 (63.64)
Progesterone receptor status, *n* (%) ^1^					
Negative	70 (43.75)	19 (38.00)	21 (43.75)	17 (42.50)	16 (48.48)
Positive	87 (54.38)	29 (58.00)	26 (54.17)	22 (55.00)	17 (51.52)
Energy intake, mea*n* ± SD (kcal/day)	1749.59 ± 380.37	1676.96 ± 319.93	1871.90 ± 413.24	1779.77 ± 375.23	1796.31 ± 435.29
Dietary supplement use, *n* (%)					
No	56 (35.00)	17 (34.00)	12 (25.00)	17 (42.50)	8 (24.24)
Yes	104 (65.00)	33 (66.00)	36 (75.00)	23 (57.50)	25 (75.76)
Alcohol intake, *n* (%) ^1^					
Never drinker	76 (47.50)	28 (56.00)	24 (50.00)	19 (47.50)	15 (45.45)
Ever drinker	83 (51.88)	22 (44.00)	23 (47.92)	21 (52.50)	18 (54.55)
Smoking status, *n* (%) ^1^					
Never smoker	139 (86.88)	45 (90.00)	36 (75.00)	36 (90.00)	26 (78.79)
Ever smoker	5 (3.13)	1 (2.00)	2 (4.17)	2 (5.00)	1 (3.03)

Abbreviations: ACS, American Cancer Society; WCRF/AICR, World Cancer Research Fund/American Institute for Cancer Research; SD, standard deviation; MET, metabolic equivalent task; AJCC, American Joint Committee on Cancer; ^1^ Number of participants was less than 160 because some participants did not provide relevant information.

**Table 4:** Health-related quality of life (HRQoL) scores according to ACS guidelines adherence score among breast cancer survivors with stage I to III (*n* = 160) ^1^.

		ACS Guidelines Score				
HRQOL Items	*n*	Q1 (*n* = 50)	Q2 (*n* = 32)	Q3 (*n* = 30)	Q4 (*n* = 48)	*p* for Trend ^2^
ACS score, range	160	0–2	3	4	5–6	
EORTC QLQ-C30, LS means (95% CI)						
Global health status/QoL	134	23.36 (15.32–35.62)	30.73 (18.74–50.39)	33.10 (19.08–57.44)	25.19 (16.00–39.67)	0.68
Functioning						
Physical Functioning	158	75.67 (65.17–87.87)	77.87 (65.48–92.59)	83.99 (69.25–101.86)	76.60 (65.13–90.09)	0.74
Role Functioning	160	63.64 (48.26–83.92)	79.45 (56.88–110.98)	105.76 (72.86–153.52)	81.32 (59.63–110.89)	0.07
Emotional Functioning	160	71.80 (58.30–88.43)	73.83 (57.41–94.96)	60.06 (45.36–79.51)	79.72 (63.11–100.70)	0.67
Cognitive Functioning	160	72.15 (61.40–84.79)	71.48 (58.82–86.86)	88.74 (71.40–110.29)	66.52 (55.51–79.72)	0.77
Social Functioning	160	57.16 (46.21–70.69)	64.57 (49.95–83.48)	77.86 (58.47–103.67)	71.06 (55.99–90.19)	0.05
Symptom						
Fatigue	159	27.30 (20.25–36.79)	25.76 (18.12–36.62)	19.77 (13.35–29.28)	24.65 (17.74–34.24)	0.37
Nausea and vomiting	160	2.71 (1.59–4.62)	2.93 (1.54–5.57)	2.35 (1.15–4.82)	2.60 (1.43–4.73)	0.79
Pain	159	10.26 (5.74–18.35)	15.99 (8.06–31.72)	6.79 (3.16–14.59)	8.38 (4.42–15.89)	0.28
Dyspnea	158	4.84 (2.53–9.24)	4.53 (2.11–9.71)	4.20 (1.80–9.84)	3.32 (1.63–6.78)	0.32
Insomnia	158	12.66 (6.94–23.09)	17.57 (8.65–35.69)	18.25 (8.27–40.28)	21.30 (10.95–41.44)	0.14
Loss of appetite	158	2.48 (1.34–4.59)	2.27 (1.10–4.68)	3.08 (1.37–6.93)	1.89 (0.96–3.73)	0.59
Constipation	158	6.92 (3.62–13.21)	5.00 (2.34–10.65)	6.96 (2.99–16.20)	6.48 (3.19–13.14)	0.99
Diarrhea	160	3.83 (2.14–6.84)	2.73 (1.35–5.50)	2.76 (1.26–6.03)	2.93 (1.53–5.62)	0.45
Financial impact	160	9.73 (5.17–18.29)	7.18 (3.35–15.40)	9.18 (3.92–21.49)	5.70 (2.81–11.57)	0.22
EORTC QLQ-BR23, LS means (95% CI)						
Functioning						
Body image	160	42.54 (27.15–66.64)	32.57 (18.94–56.01)	48.20 (26.33–88.24)	29.92 (18.08–49.50)	0.35
Sexual functioning	153	1.91 (1.03–3.55)	2.70 (1.28–5.67)	2.36 (1.05–5.30)	4.01 (2.00–8.03)	0.06
Future perspective	160	36.99 (21.71–63.02)	27.65 (14.53–52.64)	43.67 (21.30–89.53)	32.90 (18.10–59.82)	0.95
Symptom						
Systematic therapy side effects	160	21.95 (16.30–29.55)	25.72 (17.95–36.84)	19.12 (12.80–28.54)	18.89 (13.53–26.36)	0.26
Breast symptoms	160	14.19 (8.71–23.11)	12.32 (6.83–22.21)	10.31 (5.35–19.89)	11.08 (6.41–19.14)	0.34
Arm symptoms	160	19.25 (12.92–28.68)	25.87 (15.99–41.87)	20.03 (11.71–34.26)	28.65 (18.32–44.79)	0.17
Upset by hair loss	101	23.78 (11.61–48.74)	19.30 (8.45–44.07)	18.83 (6.41–55.36)	42.87 (18.83–97.59)	0.20

Abbreviations: ACS, American Cancer Society; LS means, least-squares means; 95% CI, 95% confidence interval; EORTC QLQ-C30, European Organization for Research and Treatment of Cancer Quality of Life Questionnaire Core 30; BR23, breast cancer module 23; ^1^ Models were adjusted for age (year; continuous), energy intake (kcal/day; continuous), dietary supplement use (yes, no), education level (high school or below, college or above), marital status (married or cohabitation, unmarried or divorced or widowed), breast cancer stage (I, II, III), and time since surgery (6 month-1, 1–5, ≥5 years); ^2^ *p* for trend was calculated using the median value of each quartile category as a continuous variable.

Breast cancer survivors who had higher adherence to WCRF/AICR recommendations had significantly higher scores of arm symptoms (*p* for trend = 0.01) and tended to have higher scores of insomnia (*p* for trend = 0.05) compared to those with lower adherence to WCRF/AICR recommendations (Table 5). When we examined the relationship between WCRF/AICR recommendation adherence and HRQoL according to the stage at diagnosis (stages I and II-III), significant associations were not observed between HRQoL scores and the level of adherence to WCRF/AICR recommendations among stage I breast cancer survivors (Table S1). Breast cancer survivors at breast cancer stage II or III with higher adherence to WCRF/AICR recommendations had significantly higher arm symptom scores (*p* for trend = 0.01) and higher scores for upset by hair loss (*p* for trend = 0.03) compared to those with lower adherence to WCRF/AICR recommendations (Table S2).

**Table 5:** Health-related quality of life (HRQoL) scores according to WCRF/AICR recommendation adherence score among breast cancer survivors with stage I to III (*n* = 160) ^1^.

		WCRF/AICR Recommendation Score				
HRQOL Items	*n*	Q1 (*n* = 40)	Q2 (*n* = 46)	Q3 (*n* = 41)	Q4 (*n* = 33)	*p* for Trend ^2^
WCRF/AICR score, range	160	1–4	5–6	7–8	9–12	
EORTC QLQ-C30, LS means (95% CI)						
Global health status/QoL	134	28.10 (18.45–42.79)	24.41 (14.76–40.38)	24.46 (15.37–38.92)	30.15 (17.70–51.35)	0.90
Functioning						
Physical Functioning	158	76.29 (65.68–88.62)	82.08 (69.05–97.58)	78.00 (66.15–91.98)	73.95 (61.33–89.16)	0.63
Role Functioning	160	68.05 (50.95–90.89)	84.26 (60.64–117.08)	77.20 (56.02–106.39)	84.76 (58.87–122.05)	0.44
Emotional Functioning	160	70.61 (57.00–87.47)	80.71 (63.27–102.94)	75.44 (59.50–95.63)	62.86 (48.00–82.32)	0.41
Cognitive Functioning	160	70.72 (59.80–83.63)	77.45 (64.01–93.71)	74.37 (61.76–89.56)	64.81 (52.46–80.05)	0.46
Social Functioning	160	54.17 (43.64–67.25)	77.34 (60.49–98.89)	68.10 (53.59–86.54)	76.71 (58.42–100.73)	0.09
Symptom						
Fatigue	159	24.81 (18.36–33.52)	22.30 (15.67–31.74)	27.73 (19.81–38.80)	25.92 (17.69–37.97)	0.45
Nausea and vomiting	160	2.67 (1.55–4.62)	2.41 (1.29–4.48)	2.76 (1.51–5.05)	3.09 (1.55–6.14)	0.61
Pain	159	11.07 (6.13–19.99)	8.51 (4.25–17.02)	11.01 (5.69–21.30)	8.50 (4.02–18.00)	0.81
Dyspnea	158	4.86 (2.55–9.29)	3.62 (1.69–7.73)	4.63 (2.25–9.54)	2.65 (1.17–6.03)	0.39
Insomnia	158	11.98 (6.59–21.78)	18.84 (9.34–38.00)	18.97 (9.73–36.99)	29.80 (13.87–64.04)	0.05
Loss of appetite	158	2.12 (1.15–3.92)	2.78 (1.36–5.71)	2.94 (1.48–5.83)	1.49 (0.68–3.27)	0.61
Constipation	158	6.99 (3.67–13.31)	3.64 (1.72–7.69)	7.45 (3.65–15.19)	6.33 (2.82–14.22)	0.52
Diarrhea	160	4.35 (2.41–7.86)	2.31 (1.18–4.53)	2.39 (1.24–4.59)	3.52 (1.67–7.42)	0.57
Financial impact	160	9.56 (5.00–18.26)	5.68 (2.72–11.86)	8.70 (4.24–17.83)	5.44 (2.40–12.30)	0.54
EORTC QLQ-BR23, LS means (95% CI)						
Functioning						
Body image	160	37.58 (23.91–59.07)	41.99 (25.12–70.21)	44.50 (26.96–73.45)	19.70 (11.15–34.83)	0.13
Sexual functioning	153	1.74 (0.92–3.27)	3.57 (1.73–7.37)	3.86 (1.93–7.71)	2.23 (1.03–4.84)	0.42
Future perspective	160	39.37 (22.84–67.85)	40.48 (21.80–75.15)	28.91 (15.81–52.86)	23.83 (12.00–47.31)	0.10
Symptom						
Systematic therapy side effects	160	24.14 (17.78–32.77)	17.69 (12.50–25.04)	20.63 (14.70–28.95)	21.59 (14.69–31.73)	0.86
Breast symptoms	160	13.62 (8.26–22.47)	11.63 (6.59–20.55)	10.44 (6.00–18.18)	15.16 (8.07–28.48)	0.99
Arm symptoms	160	18.09 (12.07–27.13)	22.70 (14.32–35.98)	27.16 (17.34–42.55)	35.55 (21.34–59.23)	0.01
Upset by hair loss	101	24.21 (11.96–48.97)	11.95 (4.80–29.77)	29.39 (12.72–67.89)	39.41 (16.07–96.68)	0.10

Abbreviations: WCRF/AICR, World Cancer Research Fund/American Institute for Cancer Research; LS means, least-squares means; 95% CI, 95% confidence interval; EORTC QLQ-C30, European Organization for Research and Treatment of Cancer Quality of Life Questionnaire Core 30; BR23, breast cancer module 23; ^1^ Models were adjusted for age (year; continuous), energy intake (kcal/day; continuous), dietary supplement use (yes, no), education level (high school or below, college or above), marital status (married or cohabitation, unmarried or divorced or widowed), breast cancer stage (I, II, III), and time since surgery (6 month-1, 1–5, ≥5 years); ^2^ *p* for trend was calculated using the median value of each quartile category as a continuous variable.

When we examined the association between adherence to ACS or WCRF/AIR guidelines and HRQoL levels according to the time since surgery (<1.9 or ≥1.9 years, median), social functioning scores tended to increase with increasing scores of adherence to ACS guidelines (*p* for trend = 0.05) or increasing scores of adherence to WCRF/AICR recommendations (*p* for trend = 0.04) among those who had 1.9 or more years since surgery. Among breast cancer survivors who had less than 1.9 years since surgery, those who had higher adherence to WCRF/AICR recommendations tended to have higher arm symptoms scores (*p* for trend = 0.05) compared to those who had lower adherence to WCRF/AICR recommendations (Data not shown).

## 4. Discussion

We examined the association between adherence to guidelines for cancer survivors and HRQoL among 160 Korean breast cancer survivors. High adherence to ACS guidelines was associated with increasing scores of social functioning, whereas adherence to WCRF/AICR recommendations was significantly associated with the higher scores of arm symptoms. Our findings may suggest that breast cancer survivors who had healthy dietary habits could have favorable social functioning once they manage to cope with cancer, but those with worse symptoms could try to have healthy diet partly because of some emotional anxiety. More apparent associations for social functioning among breast cancer survivors with a longer time since surgery and for arm symptoms among those with a short time since surgery may partly support our explanations. It warrants further prospective study, where a clear temporal relationship can be assessed. When we examined whether our findings differed by the stage at diagnosis, the associations for social functioning and arm symptoms were more pronounced in breast cancer survivors at stage II or III. For breast cancer survivors diagnosed with breast cancer stage I, increasing adherence to ACS guidelines was associated with enhanced physical functioning scores. It is possible that physical functioning could be an indicator of quality of life among those with early stage, and social functioning and arm symptoms may be important factors related to or trigger healthy diet among those with stage II or III.

Our finding of the association between social functioning and healthy diet corresponded to findings of a previous cross-sectional study nested in a breast cancer survivorship cohort, the Health, Eating, Activity, and Lifestyle (HEAL) study [34]. That study examined the association between HRQoL and diet quality using the Diet Quality index and found better scores of physical functioning, bodily pain, social functioning, role-emotional, and mental health subscales with higher diet quality compared to the those with poor diet quality among breast cancer survivors [34]. Several epidemiologic studies that examined overall or sub-scaled HRQoL found significant better HRQoL scores with increasing adherence to guidelines. Among the participants of ACS’s Study of Cancer Survivors-II, breast cancer survivors who met the recommendations, (1) accumulated at least 150 min of moderate-to-strenuous or 60 min of strenuous physical activity per week; (2) consumed at least five servings of fruits and vegetables each day (5-A-Day); and (3) did not smoke, had significantly better overall HRQoL scores compared to those who did not follow the recommendations [15]. The Iowa Women’s Health Study (IWHS) examined the relationship between adherence to WCRF/AICR recommendations and summary scores of the physical and mental components of SF-36 among elderly female cancer survivors and found that higher adherence scores to the recommendations were significantly associated with higher physical or mental summary scores among breast cancer survivors [17]. Another cross-sectional study found that breast, prostate, and colorectal cancer survivors with healthier diet quality had better scores of physical quality of life [35].

Our study found that those who had higher adherence to WCRF/AICR recommendations had higher scores of arm symptoms compared to those with lower adherence, however, four cross-sectional studies aforementioned did not find positive association between pain and adherence to guidelines. Differences between our study and previous studies may be partly because time since diagnosis was relatively shorter in our population than other study populations. We found that this association existed among only breast cancer survivors with a short time since surgery, but not among those with a relatively longer time since surgery. It is possible that emotional anxiety, which could be stimulated by pain or symptoms, promoted adherence to healthy diet among those who had a relatively short time since diagnosis. There is a report that breast cancer survivors who initiated dietary changes after diagnosis were more likely to have psychological distress than those who did not [36]. Further larger studies are needed to examine differences in the association between diet and HRQoL levels by time since diagnosis or levels of emotional anxiety.

For body mass index and physical activity, there is evidence that maintaining healthy weight and being physically active improved quality of life. A recent meta-analysis of 25 physical exercise intervention trials on quality of life of breast cancer survivors reported increased overall quality of life in the intervention group compared to control group [37]. Short-term intervention studies of breast cancer survivors have reported improvements in HRQoL levels with exercise and dietary intervention [38,39,40]. The Lifestyle Intervention in Adjuvant Treatment of Early Breast Cancer (LISA) study found that individualized lifestyle intervention groups had significantly higher improvement in physical HRQoL compared to the mail-based intervention group [41]. Although we cannot infer a causal relationship in this cross-sectional study, our findings may suggest the potential benefit of healthy lifestyle, involving normal body weight, physical activity and healthy diet for Korean breast cancer survivors. Further investigation with larger number of Korean breast cancer survivors is needed to provide evidence-based lifestyle guidelines for breast cancer survivors.

This is the first study to examine the association between the AICR guideline or WCRF/AICR recommendation adherence and HRQoL among Korean breast cancer survivors. Other strengths of our study include dietary assessment by three-day dietary records, which is regarded as the gold standard, and good medical information related to breast cancer diagnosis and treatment. However, we do not have information on the comorbidity status at diagnosis, which has been suggested as a predictor of impaired overall quality of life as well as functional status [42,43], and we could not determine the causal relationship between the ACS guideline and WCRF/AICR recommendation adherence and HRQoL because this is a cross-sectional study. Our findings may not be generalizable to all Korean breast cancer survivors because of the small sample size and their comparably higher level of education. Although the ACS and WCRF/AICR guidelines are widely used to estimate the overall lifestyle of the general population and cancer survivors, its scoring methods can involve arbitrary decisions, especially with regard to diet adherence scores.

## 5. Conclusions

In conclusion, we identified that adherence to ACS guidelines was associated with better social functioning scales, and this association was more pronounced among survivors with stage II or III cancer. We found that adherence to WCRF/AIRCR recommendations was associated with worse arm symptoms scales. Our results suggest that although ACS and WCRF/AICR guideline adherence by cancer survivors is associated with the status of health-related quality of life among breast cancer survivors, it may differ according to the breast cancer stage or the time since surgery. Although our study does not directly infer a causal relationship, significant association between lifestyle factors and quality of life observed in our study emphasizes the need of Korean breast cancer survivorship studies that examine the role of diet, physical activity, and other lifestyle factors in the progression of breast cancer.

## Supplementary Materials

Supplementary materials can be accessed at: http://www.mdpi.com/2072-6643/7/12/5532/s1.
