# Supplementary material for: Adherence to Guidelines for Cancer Survivors and Health-Related Quality of Life among Korean Breast Cancer Survivors
Source: Nutrients. 2015 Dec 9;7(12):10307–19. doi: 10.3390/nu7125532 (PMC4690084; doi:10.3390/nu7125532)
Supplement: Supplementary file 1 [file nutrients-07-05532-s001.docx]

Supplementary Material: Adherence to Guidelines for Cancer Survivors and Health-Related Quality of Life among Korean Breast Cancer Survivors

Sihan Song, Eunkyung Hwang, Hyeong-Gon Moon, Dong-Young Noh and Jung Eun Lee

**Table S1.** Health-related quality of life (HRQoL) scores according to ACS or WCRF/AICR guidelines adherence score among breast cancer survivors with stage I (*n* = 73) ^1^.

|  |  | **ACS Guidelines Score** | | | **WCRF/AICR Recommendation Score** | | |
| --- | --- | --- | --- | --- | --- | --- | --- |
| **HRQoL Items** | ***n*** | **Q1 (*n* =21)** | **Q4 (*n* = 24)** | ***p* for Trend ^2^** | **Q1 (*n* = 20)** | **Q4 (*n* = 16)** | ***p* for Trend ^2^** |
| Adherence score, range | 73 | 0–2 | 5–6 |  | 2–4 | 9–11 |  |
| EORTC QLQ-C30, LS means (95% CI) |  |  |  |  |  |  |  |
| Global health status/QoL | 65 | 31.57 (17.28–57.68) | 26.45 (14.98–46.72) | 0.79 | 33.68 (19.42–58.41) | 23.97 (12.38–46.42) | 0.61 |
| Functioning |  |  |  |  |  |  |  |
| Physical Functioning | 73 | 67.21 (60.06–75.22) | 81.36 (73.33–90.28) | 0.01 | 72.09 (64.48–80.61) | 82.63 (72.41–94.29) | 0.07 |
| Role Functioning | 73 | 52.04 (38.04–71.20) | 76.69 (57.40–102.46) | 0.07 | 59.41 (43.74–80.70) | 90.86 (63.26–130.50) | 0.08 |
| Emotional Functioning | 73 | 74.56 (49.16–113.06) | 75.90 (51.66–111.52) | 0.80 | 69.97 (46.50–105.29) | 48.63 (29.99–78.83) | 0.30 |
| Cognitive Functioning | 73 | 77.65 (65.57–91.96) | 70.99 (60.72–83.00) | 0.54 | 69.28 (59.00–81.35) | 69.78 (57.71–84.38) | 0.99 |
| Social Functioning | 73 | 73.46 (58.36–92.47) | 74.31 (60.07–91.92) | 0.88 | 68.73 (55.51–85.10) | 80.69 (62.68–103.88) | 0.64 |
| Symptom |  |  |  |  |  |  |  |
| Fatigue | 73 | 31.42 (19.95–49.49) | 30.38 (19.96–46.22) | 0.77 | 30.72 (19.99–47.20) | 36.64 (22.05–60.90) | 0.55 |
| Nausea and vomiting | 73 | 2.87 (1.21–6.82) | 3.70 (1.66–8.22) | 0.92 | 4.44 (1.97–9.96) | 6.13 (2.36–15.96) | 0.85 |
| Pain | 73 | 12.93 (5.41–30.91) | 13.77 (6.16–30.81) | 0.94 | 16.00 (7.06–36.25) | 16.11 (6.13–42.37) | 0.96 |
| Dyspnea | 72 | 2.94 (1.08–8.00) | 3.85 (1.53–9.68) | 0.76 | 5.57 (2.17–14.31) | 3.80 (1.25–11.51) | 0.64 |
| Insomnia | 73 | 17.75 (7.08–44.53) | 20.53 (8.78–48.02) | 0.71 | 15.85 (6.66–37.73) | 27.98 (10.04–78.01) | 0.33 |
| Loss of appetite | 73 | 2.76 (1.02–7.47) | 2.22 (0.89–5.57) | 0.82 | 2.19 (0.85–5.62) | 1.97 (0.65–6.01) | 0.99 |
| Constipation | 73 | 6.52 (2.38–17.86) | 10.95 (4.31–27.79) | 0.33 | 8.43 (3.24–21.97) | 7.86 (2.53–24.40) | 0.80 |
| Diarrhea | 73 | 3.29 (1.34–8.03) | 2.75 (1.21–6.29) | 0.86 | 4.53 (1.98–10.39) | 5.05 (1.89–13.45) | 0.85 |
| Financial impact | 73 | 6.71 (2.62–17.18) | 5.65 (2.37–13.46) | 0.73 | 7.46 (2.98–18.68) | 6.97 (2.35–20.64) | 0.96 |
| EORTC QLQ-BR23, LS means (95% CI) |  |  |  |  |  |  |  |
| Functioning |  |  |  |  |  |  |  |
| Body image | 73 | 56.38 (30.16–105.37) | 43.98 (24.67–78.39) | 0.67 | 43.89 (24.53–78.54) | 32.02 (16.10–63.71) | 0.63 |
| Sexual functioning | 71 | 4.55 (1.70–12.20) | 4.67 (1.85–11.82) | 0.98 | 3.99 (1.56–10.20) | 3.04 (1.00–9.26) | 0.74 |
| Future perspective | 73 | 25.09 (9.93–63.40) | 30.83 (13.09–72.63) | 0.90 | 29.84 (12.55–70.99) | 18.92 (6.79–52.71) | 0.39 |

**Table S1.** *Cont.*

|  |  | **ACS Guidelines Score** | | | **WCRF/AICR Recommendation Score** | | |
| --- | --- | --- | --- | --- | --- | --- | --- |
| **HRQoL Items** | ***n*** | **Q1 (*n* =21)** | **Q4 (*n* = 24)** | ***p* for Trend ^2^** | **Q1 (*n* = 20)** | **Q4 (*n* = 16)** | ***p* for Trend ^2^** |
| Symptom |  |  |  |  |  |  |  |
| Systematic therapy side effects | 73 | 25.23 (16.50–38.59) | 22.66 (15.30–33.56) | 0.67 | 30.98 (21.14–45.39) | 23.11 (14.71–36.30) | 0.69 |
| Breast symptoms | 73 | 10.34 (5.26–20.32) | 13.55 (7.26–25.30) | 0.38 | 11.71 (6.28–21.83) | 18.16 (8.70–37.91) | 0.37 |
| Arm symptoms | 73 | 14.10 (8.16–24.37) | 20.11 (12.13–33.35) | 0.19 | 18.01 (10.84–29.95) | 19.44 (10.66–35.46) | 0.41 |
| Upset by hair loss | 47 | 27.96 (8.89–87.95) | 35.79 (10.97–116.78) | 0.39 | 20.62 (7.29–58.34) | 30.54 (8.33–112.00) | 0.40 |

Abbreviations: ACS, American Cancer Society; WCRF/AICR, World Cancer Research Fund/American Institute for Cancer Research; LS means, least-squares means; 95% CI, 95% confidence interval; EORTC QLQ-C30, European Organization for Research and Treatment of Cancer Quality of Life Questionnaire Core 30; BR23, breast cancer module 23; ^1^ Models were adjusted for age (year; continuous), energy intake (kcal/day; continuous), dietary supplement use (yes, no), education level (high school or below, college or above), marital status (married or cohabitation, unmarried or divorced or widowed), time since surgery (6 month-1, 1–5, ≥5 years); ^2^ *p* for trend was calculated using the median value of each quartile category as a continuous variable.

**Table S2.** Health-related quality of life (HRQoL) scores according to ACS or WCRF/AICR guidelines adherence score among breast cancer survivors with stage II or III
(*n* = 87) ^1^.

|  |  | **ACS Guidelines Score** | | | **WCRF/AICR Recommendation Score** | | |
| --- | --- | --- | --- | --- | --- | --- | --- |
| **HRQoL Items** | ***n*** | **Q1 (*n* = 29)** | **Q4 (*n* = 24)** | ***p* for Trend ^2^** | **Q1 (*n* = 20)** | **Q4 (*n* = 17)** | ***p* for Trend ^2^** |
| Adherence score, range | 87 | 0–2 | 5–6 |  | 1–4 | 9–12 |  |
| EORTC QLQ-C30, LS means (95% CI) |  |  |  |  |  |  |  |
| Global health status / QoL | 69 | 19.38 (10.14–37.06) | 25.33 (12.34–52.01) | 0.39 | 23.07 (11.66–45.65) | 41.67 (17.33–100.19) | 0.52 |
| Functioning |  |  |  |  |  |  |  |
| Physical Functioning | 85 | 79.83 (60.98–104.50) | 71.28 (51.90–97.90) | 0.63 | 79.38 (60.12–104.81) | 67.21 (47.00–96.11) | 0.26 |
| Role Functioning | 87 | 72.61 (46.24–114.03) | 83.30 (47.64–145.67) | 0.34 | 75.43 (46.07–123.50) | 76.24 (40.04–145.18) | 0.91 |
| Emotional Functioning | 87 | 65.49 (56.44–76.00) | 73.14 (60.83–87.94) | 0.16 | 64.99 (55.55–76.05) | 78.30 (63.78–96.14) | 0.57 |
| Cognitive Functioning | 87 | 71.47 (55.00–92.86) | 62.41 (45.12–86.32) | 0.97 | 71.97 (54.01–95.90) | 56.67 (38.95–82.46) | 0.41 |
| Social Functioning | 87 | 50.32 (35.76–70.81) | 69.48 (45.52–106.06) | 0.05 | 45.21 (31.37–65.16) | 70.84 (43.95–114.21) | 0.14 |

**Table S2.** *Cont.*

|  |  | **ACS Guidelines Score** | | | **WCRF/AICR Recommendation Score** | | |
| --- | --- | --- | --- | --- | --- | --- | --- |
| **HRQoL Items** | ***n*** | **Q1 (*n* = 29)** | **Q4 (*n* = 24)** | ***p* for Trend ^2^** | **Q1 (*n* = 20)** | **Q4 (*n* = 17)** | ***p* for Trend ^2^** |
| Symptom |  |  |  |  |  |  |  |
| Fatigue | 86 | 27.38 (18.35–40.85) | 20.67 (12.76–33.49) | 0.13 | 26.12 (17.25–39.56) | 19.20 (11.07–33.28) | >0.99 |
| Nausea and vomiting | 87 | 2.54 (1.28–5.05) | 2.05 (0.87–4.79) | 0.58 | 2.12 (1.02–4.42) | 2.22 (0.85–5.78) | 0.50 |
| Pain | 86 | 8.61 (3.89–19.07) | 5.10 (1.96–13.31) | 0.19 | 8.71 (3.67–20.65) | 5.40 (1.72–16.98) | 0.98 |
| Dyspnea | 86 | 5.22 (2.23–12.20) | 3.04 (1.09–8.48) | 0.28 | 4.04 (1.69–9.64) | 2.46 (0.78–7.80) | 0.63 |
| Insomnia | 85 | 11.61 (5.16–26.10) | 25.16 (9.38–67.43) | 0.18 | 12.02 (5.18–27.91) | 36.76 (11.76–114.96) | 0.14 |
| Loss of appetite | 85 | 2.67 (1.23–5.78) | 1.91 (0.74–4.90) | 0.53 | 2.93 (1.33–6.46) | 1.28 (0.44–3.72) | 0.21 |
| Constipation | 85 | 8.21 (3.43–19.67) | 4.51 (1.58–12.83) | 0.38 | 7.77 (3.16–19.12) | 5.39 (1.66–17.57) | 0.74 |
| Diarrhea | 87 | 4.02 (1.81–8.94) | 3.86 (1.44–10.37) | 0.63 | 3.91 (1.64–9.36) | 2.79 (0.89–8.72) | 0.73 |
| Financial impact | 87 | 11.13 (4.73–26.18) | 5.52 (1.91–15.92) | 0.10 | 10.84 (4.29–27.38) | 4.12 (1.23–13.81) | 0.50 |
| EORTC QLQ-BR23, LS means (95% CI) |  |  |  |  |  |  |  |
| Functioning |  |  |  |  |  |  |  |
| Body image | 87 | 28.89 (15.38–54.27) | 19.18 (8.78–41.87) | 0.42 | 33.73 (17.46–65.15) | 11.58 (4.90–27.38) | 0.06 |
| Sexual functioning | 82 | 1.06 (0.46–2.41) | 3.89 (1.39–10.93) | 0.01 | 0.84 (0.34–2.06) | 1.97 (0.65–5.95) | 0.11 |
| Future perspective | 87 | 44.21 (23.57–82.89) | 28.19 (12.94–61.41) | 0.68 | 49.37 (24.32–100.24) | 23.58 (9.35–59.48) | 0.08 |
| Symptom |  |  |  |  |  |  |  |
| Systematic therapy side effects | 87 | 22.43 (14.82–33.93) | 17.45 (10.45–29.14) | 0.25 | 22.08 (13.97–34.91) | 24.83 (13.65–45.16) | 0.75 |
| Breast symptoms | 87 | 18.79 (9.21–38.32) | 8.99 (3.72–21.72) | 0.08 | 15.74 (7.23–34.29) | 12.58 (4.55–34.78) | 0.57 |
| Arm symptoms | 87 | 24.20 (13.52–43.31) | 39.26 (19.09–80.72) | 0.40 | 17.50 (9.61–31.84) | 76.26 (34.88–166.75) | 0.01 |
| Upset by hair loss | 54 | 16.82 (6.22–45.52) | 59.97 (18.67–192.59) | 0.17 | 19.93 (7.09–56.03) | 70.62 (17.43–286.08) | 0.03 |

Abbreviations: ACS, American Cancer Society; WCRF/AICR, World Cancer Research Fund/American Institute for Cancer Research; LS means, least-squares means; 95% CI, 95% confidence interval; EORTC QLQ-C30, European Organization for Research and Treatment of Cancer Quality of Life Questionnaire Core 30; BR23, breast cancer module 23; ^1^ Models were adjusted for age (year; continuous), energy intake (kcal/day; continuous), dietary supplement use (yes, no), education level (high school or below, college or above), marital status (married or cohabitation, unmarried or divorced or widowed), breast cancer stage III (yes, no), and time since surgery (6 month-1, 1–5, ≥5 years); ^2^ *p* for trend was calculated using the median value of each quartile category as a continuous variable.
